# Episodic Severe Ectopic Adrenocorticotropic Hormone Syndrome by Metastatic Appendiceal Neuroendocrine Tumor

**DOI:** 10.1210/jcemcr/luaf171

**Published:** 2025-08-01

**Authors:** Ruchi Desai, F N U Sidra, Liwei Jia, Patricio M Polanco, Salwan Al Mutar, Oksana Hamidi

**Affiliations:** Department of Internal Medicine, University of Texas Southwestern Medical Center, Dallas, TX 75390, USA; Division of Endocrinology and Metabolism, University of Texas Southwestern Medical Center, Dallas, TX 75390, USA; Division of Pathology, University of Texas Southwestern Medical Center, Dallas, TX 75390, USA; Division of Surgical Oncology, University of Texas Southwestern Medical Center, Dallas, TX 75390, USA; Division of Hematology/Oncology, University of Texas Southwestern Medical Center, Dallas, TX 75390, USA; Division of Endocrinology and Metabolism, University of Texas Southwestern Medical Center, Dallas, TX 75390, USA

**Keywords:** Cushing syndrome, Ectopic ACTH syndrome, Ectopic Cushing syndrome, appendiceal neuroendocrine tumor, severe hypercortisolism

## Abstract

Episodic severe Cushing syndrome resulting from ectopic ACTH secretion (EAS) from appendiceal neuroendocrine tumor (NET) is extremely rare. Here, we describe a case of a 24-year-old woman with episodic severe EAS resulting from appendiceal NET with extensive metastatic disease. The patient presented with rapid weight gain, violaceous striae, fatigue, edema, and anxiety. Biochemical evaluation showed markedly elevated 24-hour urinary free cortisol greater than 10-fold above the upper limit of normal, and widely fluctuating peaks and troughs of serum cortisol and ACTH concentrations indicating episodic severe EAS. Surgery for primary malignancy was initially deferred because of the high risk of perioperative complications related to severe hypercortisolism. She underwent bilateral adrenalectomy as first-line definitive treatment for severe EAS. Four months after adrenalectomy, she underwent cytoreductive surgery for primary metastatic appendiceal NET. Subsequent peptide receptor radionuclide therapy and monthly lanreotide injections rendered her disease stable. Three years after the initial presentation, she continued to undergo active surveillance with maintenance lanreotide for residual but stable metastatic appendiceal neuroendocrine tumor. This case of a rare metastatic appendiceal NET with EAS demonstrates the importance of individualized management and highlights the need for consideration of prompt bilateral adrenalectomy for patients with severe Cushing syndrome.

## Introduction

Ectopic ACTH secretion (EAS) from nonpituitary tumors is a rare cause of Cushing syndrome accounting for 5% to 10% of Cushing syndrome cases [1-4]. EAS is caused by production of ACTH from nonpituitary tumors and is most commonly seen secondary to bronchial neuroendocrine tumor (NET), followed by pancreatic NET and small cell lung cancer [2, 5, 6]. Other rare causes include thymic NET, medullary thyroid carcinoma, islet cell tumors, and pheochromocytoma [5]. Up to 8% to 19% of cases with EAS have an occult/unknown primary source [7-10]. Since 1971, only 9 cases of EAS secondary to an appendiceal NET are reported in the literature and only 3 of these cases had evidence of episodic hypercortisolism [8, 11]. Despite recent progress in Cushing syndrome therapeutics, EAS remains challenging to diagnose and manage. EAS produced by gastrointestinal NET, and more specifically appendiceal NET, is exceedingly rare, especially in the setting of metastatic disease. Here, we report a case of a patient with episodic severe EAS due to metastatic appendiceal NET managed with prompt bilateral adrenalectomy.

## Case Presentation

A 24-year-old woman with no medical history presented to her primary care clinic after developing a 60-pound weight gain, acne, generalized weakness, heat intolerance, fatigue, lower extremity swelling, palpitations, and anxiety over a 3-month period. She also reported secondary amenorrhea for 6 months before presentation. Family history was significant for bronchial NET in her mother.

## Diagnostic Assessment

**Figure 1.**
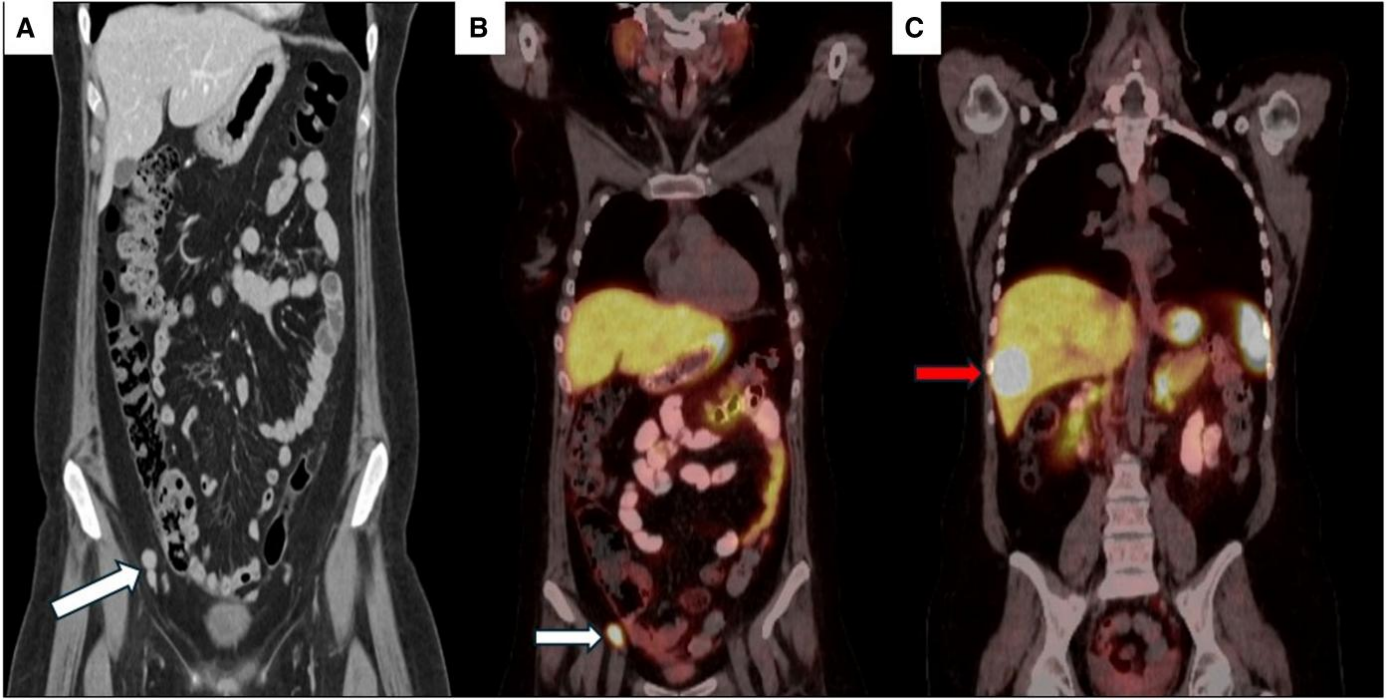
Metastatic neuroendocrine tumor imaging. (A) Coronal image from initial computed tomography of the abdomen and pelvis demonstrating a 3-cm appendiceal mass (arrow). (B and C) Coronal mages from Gallium-68 (Ga-68) DOTA-Tyr3-octreotate (DOTATATE) positron emission tomography/CT (PET/CT) redemonstrating appendiceal mass (B, arrow) and hepatic mass (C, arrow).

**Figure 2.**
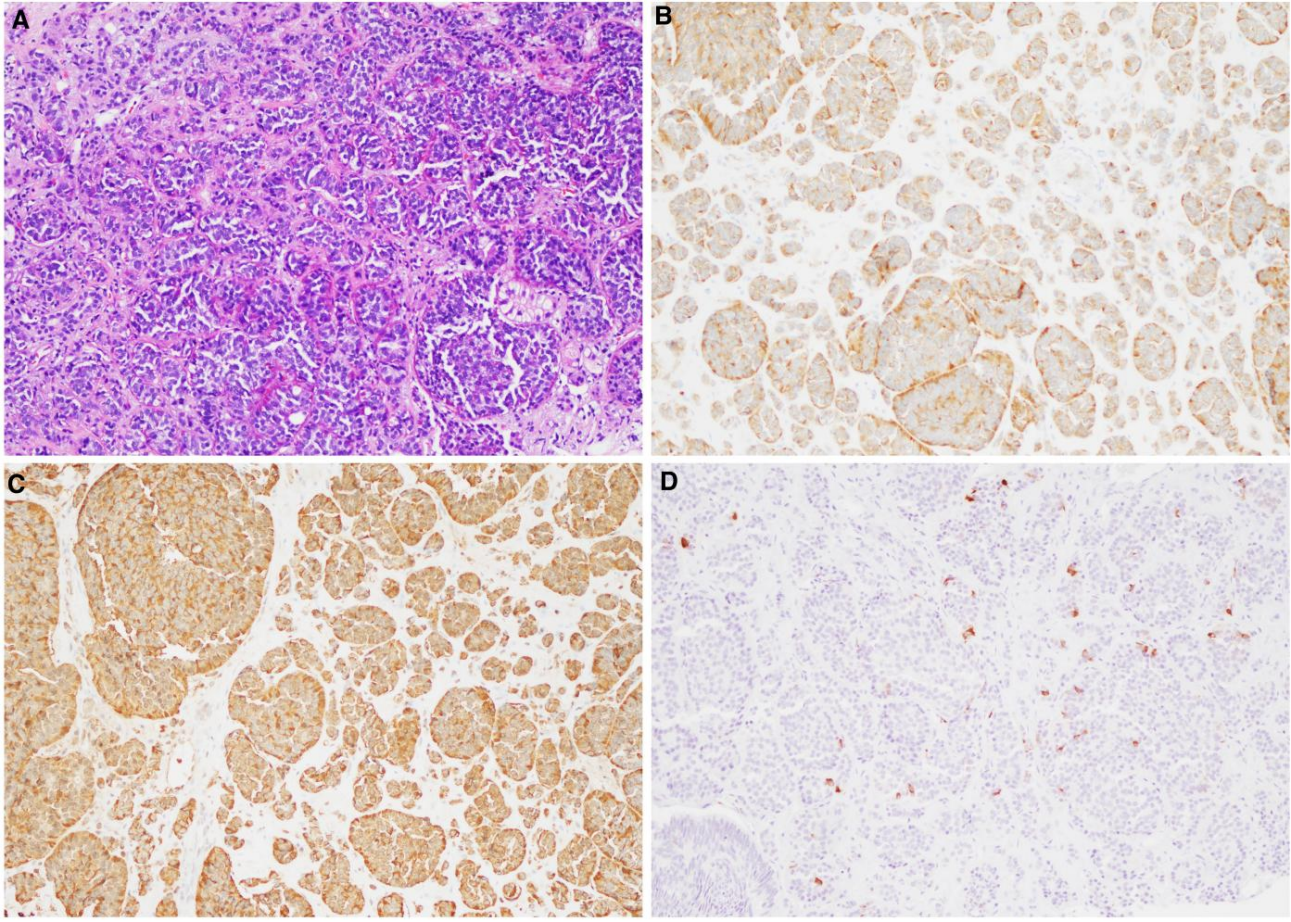
Histopathologic and immunohistochemical features of metastatic neuroendocrine tumor. Metastatic neuroendocrine tumor showed tumor cells with round or oval nuclei with salt and pepper chromatin and moderate eosinophilic granular cytoplasm arranged in nests, tubules and trabecular patterns (A), positive for synaptophysin (B) and chromogranin (C), and focally positive for ACTH (D).

On physical examination, she had moon facies, wide purple striae on her abdomen, hirsutism, and dorsocervical and supraclavicular fat pads. Her blood pressure was normal. Initial laboratory workup showed potassium 3.2 mmol/L (reference range, 3.5-5.3), 24-hour urinary free cortisol (UFC) 473 μg/24 hours (1304.5 nmol/24 hours) (reference range, <50 μg/24 hours [<138 nmol/24 hours]), ACTH 16 pg/mL (3.52 pmol/L) (reference range, 6-50 pg/mL [1.32-11 pmol/L]), and early morning serum cortisol 15.3 μg/dL (421.1 nmol/L) (reference range, 5-22 μg/dL [138-607 nmol/L]). After an overnight 1-mg dexamethasone suppression test, serum cortisol was 8.8 μg/dL (242.8 nmol/L) (reference range: ≤1.8 μg/dL [≤50 nmol/L]). Repeat 24-hour UFC 2 weeks later was 539 μg/24 hours (1486.6 nmol/24 hours). Evaluation with computed tomography (CT) of the abdomen and pelvis revealed a 3-cm enhancing appendiceal mass, enlarged peri-appendiceal mesenteric lymph nodes, and a 4-cm enhancing right hepatic lobe lesion (Fig. 1). Functional imaging with Gallium-68 DOTA-Tyr3-octreotate (DOTATATE) positron emission tomography/CT (PET/CT) demonstrated increased radiotracer uptake in the appendiceal mass, lymph nodes, hepatic mass, and right breast (Fig. 1). The pituitary gland appeared normal on magnetic resonance imaging. The patient underwent liver biopsy, and pathology showed well-differentiated grade 1 NET with positive ACTH immunohistochemistry (Fig. 2).

## Treatment

**Figure 3.**
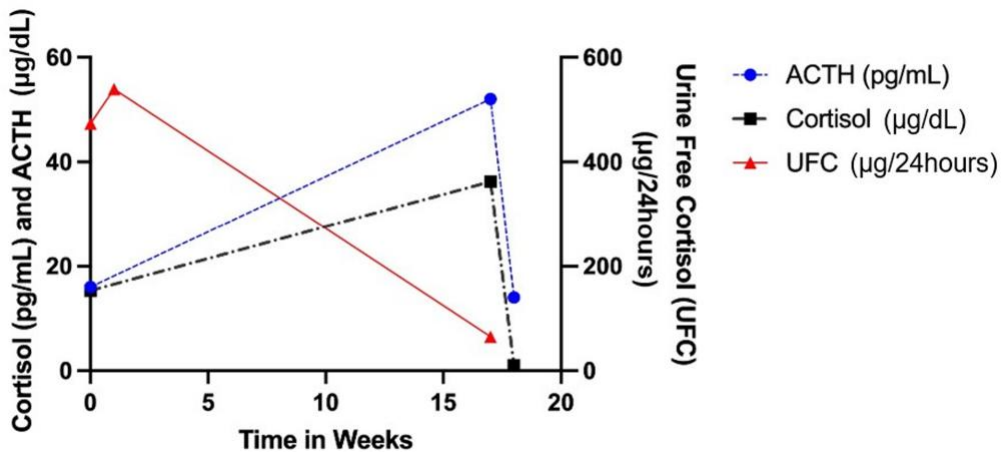
Episodic severe cortisol secretion demonstrated by serial laboratory measurements prior to bilateral adrenalectomy. ACTH (blue dashed line), serum cortisol (black dashed line), and 24-hour urine free cortisol (UFC) (red solid line) on initial presentation and before bilateral adrenalectomy. The peaks and troughs demonstrate episodic nature of cortisol production in this case.

She was subsequently referred to our center for management of metastatic NET and EAS. The patient was initially treated with ketoconazole 200 mg daily but developed insomnia and parasomnia and subsequently discontinued the medication. Repeat biochemical testing showed 24-hour UFC 65 μg/24 hours (179.3 nmol/24 hours), ACTH 52 pg/mL (11.5 pmol/L), and serum cortisol 36.2 μg/dL (999 nmol/L). Two weeks later, ACTH decreased to 14 pg/mL (3.1 pmol/L) and serum cortisol was <1 μg/dL (<27.6 nmol/L) (Fig. 3 and Table 1), concerning for episodic Cushing syndrome.

**Table 1.** Laboratory values demonstrating episodic severe Cushing syndrome

Laboratory test	Day collected	Conventional units (SI units)	Reference range
24-h UFC	0	473 µg/24 hours (1304.5 nmol/24 hours)	<50 µg/24 hours (<138 nmol/24 hours)
	20	539 µg/24 hours (1486.6 nmol/24 hours)	<50 µg/24 hours (<138 nmol/24 hours)
	101	65 µg/24 hours (179.3 nmol/24 hours)	<50 µg/24 hours(<138 nmol/24 hours)
ACTH	0	16 pg/mL (3.52 pmol/L)	6-50 pg/mL (1.32-11 pmol/L)
	101	52 pg/mL (11.5 pmol/L)	6-50 pg/mL (1.32-11 pmol/L)
	112	14 pg/mL (3.1 pmol/L)	6-50 pg/mL (1.32-11 pmol/L)
Cortisol	0	15.3 µg/dL (421.1 nmol/L)	5-22 µg/dL (138-607 nmol/L)
	20	36.2 µg/dL (999 nmol/L)	5-22 µg/dL (138-607 nmol/L)
	112	<1 µg/dL (<27.6 nmol/L)	5-22 µg/dL (138-607 nmol/L)
Cortisol after DST	16	8.8 µg/dL (242.8 nmol/L)	≤1.8 µg/dL (≤50 nmol/L)
HbA1c	78	5.1%	<5.7%

Abbreviations: DST, dexamethasone suppression test; HbA1c, hemoglobin A1c; SI, Système International (International System of Units); UFC, urinary free cortisol.

We recommended bilateral adrenalectomy given the severe episodic Cushing syndrome with extensive tumor burden in a young woman. She underwent robotic bilateral adrenalectomy and the pathology from the left adrenal gland was benign, but the right adrenal gland was notable for metastatic well differentiated NET on histologic evaluation. The patient was started on lanreotide 120 mg every 4 weeks for antiproliferative benefit. Four months after bilateral adrenalectomy, Cushingoid features markedly improved, and she successfully underwent extensive debulking surgery that included omentum resection, splenectomy, right hemicolectomy, left hemicolectomy, hysterectomy and bilateral salpingo-oophorectomy, lymphadenectomy, cholecystectomy, falciform ligament resection, liver segment 6 and 7 resection, right diaphragm mass resection, pericardial mass resection, and multiple nodules in the peritoneum. The pathology confirmed intermediate grade well-differentiated NET with Ki-67 of 5%. Comprehensive genetic testing with the CustomNext-Cancer Panel of 85 genes by Ambry Genetics was negative and DNA analysis of her tumor did not reveal any pathogenic variants.

## Outcome and Follow-up

Following debulking surgery, a repeat CT scan showed low metastatic burden, and the patient continued on lanreotide. About 1.5 years later, she developed disease progression noted on Gallium-68 DOTATATE PET/CT, demonstrating overall progressive metastatic disease with new tracer-avid periportal nodes, increased avidity of an aortocaval lymph node, and increasing size of a right external iliac node. She was started on treatment with peptide receptor radionuclide therapy and 1 year later CT of the chest, abdomen, and pelvis showed stable metastatic disease. Most recent DOTATATE PET/CT, 3 years following the initial diagnosis, showed stable residual metastatic appendiceal NET. She continues to receive maintenance monthly lanreotide injections.

## Discussion

This case demonstrates episodic severe EAS secondary to metastatic appendiceal NET managed with bilateral adrenalectomy to allow for rapid control of Cushing syndrome and subsequent tumor-targeted surgery and systemic treatments. EAS resulting from appendiceal NET is exceedingly rare, with all prior cases described in females with an age range of 15 to 35 years [11-18]. Notably, this is the only case in which a patient had extensive metastatic disease on initial presentation and the largest tumor described thus far at 3 cm in size.

Cushing syndrome in the context of EAS can be associated with intermittent episodes of ACTH and cortisol hypersecretion, interspersed with periods of normal cortisol secretion or even adrenal insufficiency. The episodes can be irregular in duration and frequency, ranging from days to months, and are unpredictable, which complicates both diagnosis and management [11, 19, 20]. The main diagnostic difficulty arises from the biochemical variability: testing during a trough may yield normal results, leading to false reassurance or missed diagnosis, whereas testing during a peak confirms hypercortisolism. This necessitates repeated and frequent biochemical assessments to capture the hypercortisolemic phase. Imaging may not localize the ectopic source, especially if the tumor is occult or small [11, 19, 20]. Diagnostic accuracy of bilateral inferior petrosal sinus sampling is significantly reduced if not performed during a hypercortisolemic phase, increasing the risk of misclassification and unnecessary surgery [11, 19].

Management of episodic severe Cushing syndrome in the setting of EAS is further complicated by the unpredictable nature of cortisol excess, which can delay both localization and definitive treatment of the ectopic source. The Endocrine Society clinical practice guidelines for Cushing syndrome recommend that first-line treatment for endogenous Cushing syndrome should be surgical removal of the tumor [21]. However, this is not always feasible, as our patient presented with metastatic disease that would require extensive debulking surgery with concern that debulking surgery alone would not control Cushing syndrome. In such cases, early medical management of hypercortisolism followed by resection of the primary tumor or bilateral adrenalectomy should be considered. Our patient was unable to tolerate ketoconazole. Alternative medical therapies were considered, but ultimately, bilateral adrenalectomy was selected given priority for timely control of hypercortisolism.

Consideration of the individual patient's risk factors for negative outcomes is important when deciding on management. A multicenter retrospective analysis of 110 patients with EAS found that the severity of hypercortisolism, hypokalemia, presence of distant metastasis, and diabetes mellitus were associated with worse prognosis [22] . In addition, 24-hour UFC greater than 5-fold above the upper limit of normal is an independent negative prognostic factor, and patients who underwent bilateral adrenalectomy had better survival rates in the first 2 years [22]. Our patient had episodic severe hypercortisolism, hypokalemia, distant metastases, and markedly elevated 24-hour UFC. By proceeding with bilateral adrenalectomy as first-line treatment, we achieved control of life-threatening Cushing syndrome. Other reported cases of EAS resulting from appendiceal NET achieved control of hypercortisolism through appendectomy ± right hemicolectomy based on the size of the appendiceal NET [11, 12]. Furthermore, our patient was a young female for whom consideration of fertility preservation was important. Proceeding with bilateral adrenalectomy and obtaining control of Cushing syndrome allowed more time to consider her fertility preservation options. This case demonstrates that management of EAS resulting from NET should be individualized, and prompt bilateral adrenalectomy should be considered when rapid control of Cushing syndrome is needed, especially in cases presenting with metastatic disease. Furthermore, this case exemplifies the importance of a multidisciplinary approach with treatment options that may only be available at a high-level cancer center.

## Learning Points

- Cushing syndrome due to ectopic ACTH secretion (EAS) by appendiceal neuroendocrine tumor is exceedingly rare with only 10 cases described in the literature to date.
- Severe Cushing syndrome is life-threatening, and management of hypercortisolism should be prompt.
- Bilateral adrenalectomy should be considered as prompt first-line treatment in those with severe uncontrolled Cushing syndrome due to EAS, when resection of the primary tumor is not feasible.

## Contributors

All authors made individual contributions to authorship. R.D. and F.S. were involved in manuscript writing. O.H., P.P., and S.M. were involved in the diagnosis and management of the patient. L.J. assisted with preparation of pathology images. All authors reviewed and approved the final draft.

O.H. is an Editorial Board Member for *JCEM Case Reports* and played no role in the journal's evaluation of the manuscript.

## Data Availability

Data sharing is not applicable to this article as no datasets were generated or analyzed during the current study.
